# Notes on Obstetrics and Gynæcology

**Published:** 1875-05

**Authors:** E. N. Brush


### Medical Notes.

ART. I.—*Notes on Obstetrics and Gynæcology.* By E. N. Brush, M. D.

1. On the Postural Treatment of Prolapsus of the Funis Umbilicalis. By John Brunton, M. D., etc. (*Obstet. Journal Great Britain and Ireland*, April, 1875.)

2. On Puerperal Fever. The Gulstonian Lectures before the Royal College of Physicians, London. By R. J. Lee, M. D., F. R. C. P. (*The London Lancet*, April, 1875; *The Doctor*, May, 1, 1875.)

3. Contributions to Obstetrics. By Theophilus Parvin, M. D. (*American Practitioner*, March, 1875.)

4. Rupture of the Perinæum. With a description of a new operation. By D. Warren Brickell, M. D. (*American Journal of Medical Sciences*, April, 1875.)

1. The reasons which the author gives for the production of this paper are four. (1) Because of the high infant mortality which is met with in prolapse of the funis, notwithstanding the ordinary treatment. (2) Because the means now employed are comparatively unknown to those daily employed in the practice of midwifery. (3) Because in most of the manuals and standard works on Obstetrics the subject of postural treatment is not mentioned, or at least, only in a cursory manner. (4) Because of the success which has attended the postural treatment of prolapse of the funis.

Mention is made of the almost invariable fatal termination, to the infant, in cases in which prolapse is allowed to continue till the delivery of the child. The cases in which the life of the child is saved, being in women who have large pelves, and easily dilatable parts, and in whom the delivery is very rapid.

The predisposing causes of prolapse are enumerated as:—1. An excessive amount of liquor amnii. 2. The same cause and a small child. 3. A wide pelvis. 4. A wide pelvis and a small child, or the presence of twins. 5. Malposition of the fœtus. 6. Deficient action of the lower segment of the uterus. 7. A narrow pelvic brim. 8 Position of the placenta near the os. 9. Length of the cord, if very long. 10. Dropsy, and therefore weight of the cord.

The exciting causes are:—1. Sudden gush of the liquor amnii when rising to the erect position. 2. Early rupture of the membrane when the child is small, premature or malpresenting.

The author does not attempt a full discussion of the subject of prolapse of the funis, but reviews the various methods which have been suggested for its correction. The earliest mention which is made of the postural treatment is by Prof. Alex. Hamilton, of Edinburgh, who advises reduction of the cord by placing the woman in the proper position; but he fails to mention the exact position. Drs. Hardy and McClintock approach the presnt method nearly, when they advise that the woman should be placed on her side opposite to that on which the cord protrudes. It remained, however, for Prof. Thomas, of New York, to suggest the true position. Upon investigating the subject, he comes to two conclusions. 1. That the cause of the persistence of this accident (whatever may at first have produced it) reduce themselves to two—the slippery nature of the displaced part, and the inclined plane offered it by the uterus by which to roll out of its cavity. 2. That the only rational mode of treatment would be inverting this plane and thus turning to our advantage not only it, but the lubricity of the cord, which ordinarily constitutes the main barrier to success. He says :*—" This, I found, could be readily accomplished *by placing the woman on her knees, with the head down upon the bed,* in the posture assumed by eastern nations in worship.

Dr. Brunton adds, that when this position cannot be readily assumed, recourse can be had to a modification of the position ad-

---

Transactions of the New-York Academy of Medicine, Vol. II., Part 2, p. 21.

vised by Drs. Hardy and McClintock, viz., by placing her on the side opposite the prolapse, and elevating the pelvis on pillows.

Ten cases are cited in conclusion, in which the cord was reduced after the manner recommended by Prof. Thomas. In eight, the children were saved, in one, the cord was pulseless before reduction, and in one a diseased placenta was present of sufficient extent to account for the death of the child.

At the conclusion of the paper, a hint is thrown out that by the employment of this position, pressure of the uterus on kidneys and renal vessels may be relieved, and uremic convulsions avoided.

2. The subject of Puerperal Fever seems just at this time to be attracting considerable attention from English obstetricians. Dr. Lee took it for his subject in the Gulstonian Lectures, and in the Obstetrical Society quite an animated discussion was had upon the same topic. In Dr. Lee's lecture no new ideas are advanced, but the lecturer has contented himself with tracing in detail the progress of the present views throgh the various stages of development.

The debate at the Obstetrical Society was opened by Mr. Spencer Wells. In his remarks he classed puerperal fever with septicæmia and pyæmia, and stated his belief to be that by proper precautions lying-in hospitals may be rid of puerperal fever just as general hospitals have been rid of pyæmia. In this view he was supported by others present, especially Dr. Leishman, who said that he renounced the opinion which he once held and had arrived at the conclusion, that puerperal fever was generally of pyæmic and septicæmic character.

3. Dr. Parvin's paper consists of a report of two cases of retroflexion of the pregnant uterus, one of placenta prævia, and one each of ruptured perinæum and of uterine inversion. In the first cases of retroflexion he first saw the patient on account of leucorrhœa, pelvic pain and rectal tenesmus. The lady had been an invalid ever since the birth of her first child, about two years previous, and at the time of consultation had not menstruated for six weeks. Manipulation was resorted to several times, and the horse-shoe pessary was also employed to restore the displacement, but without success. The uterine sound was introduced twice. In six weeks from the first consultation and a month after the last introduction of the sound the patient aborted. This result the Doctor does not think is to be regretted, as he regards it impossible to restore the uterus to its normal position. Case second was similar to the other as regards symptoms. The uterus could be readily restored by the introduction of two fingers in the rectum and one in the vagina upon the anterior portion of the cervix. The treatment employed was the frequent evacuation of the bladder, the assumption of the knee-elbow and the use of Hodge's pessary. In two months the retroflexion was gone and the uterine globe could be plainly felt in the hypogastric region.

In the case of placenta prævia, he was called to the patient on account of hemorrhage at the seventh month of pregnancy. This was restrained by the employment of the tampon, but recurred occasionally during the next two weeks, when an alarming hemorehage took place. At this he determined to induce premature labor. Dilatation was effected with Molesworth's dilators to about two inches in diameter, the finger was then passed up between the placenta and the uterus and the membranes ruptured. In two hours and a half the head was well down in the pelvis and delivery was acccomplished with the forceps. The mother and child have done well. Dr. Parvin believes that the rule asserted by Prof. Thomas is the better one to follow, "that in every case of declared placenta prævia, premature delivery should be induced," rather than the one of Leishman, to endeavor to avert premature delivery as long as possible.

In the case of ruptured perinæum the rupture extended through the perinæum and involved the recto-vaginal wall for two and one-half inches. The operation was immediate and consisted in drawing the recto-vaginal rent together by two silver sutures, and the perinæum by the same number. In eight days the sutures were removed with the restoration perfect.

The case of inversion of the uterus was one of over a year's duration. The first attempt at reduction was of two hour's duration and consisted of different modes of taxis, but little progress was made, and an attempt was made to retain it by pressing the concave end of a stethoscope against the fundus, retaining it by a T bandage. This had to be removed in twenty-four hours on account of the pain it produced. At the expiration of a week an attempt was made to employ an apparatus to make continuous elastic pressure, but this also had to be discontinued in forty-eight hours. One month after the first attempt another effeort was made by manipulation, and the employment of Prof. White's uterine repositor. This attempt was followed by success in two hours and a half after the induction of anæsthesia. The patient recovered entirely. The operator seems highly pleased with Prof. White's repositor, and expresses his preference for it over the various modes of taxis.

4. Prof. Brickell reviews the various methods of operation at present pursued for ruptured perinæum, and cites his objections to them. With the plain interrupted suture he contends that the force of apposition is antero-posterior, and that consequently the areas of denuded surface are corrogated and thus diminished, and that if union takes place it is not to the extent of surface prepared by the knife. The union which takes place is not the deep approximation of the parts, restoring the wedge-shaped perinæum, but simply the formation of an abrupt wall which serves only to close the gaping orifice. The cutting through of the wire on the posterior portion of the wound is also urged against this operation.

The same law applies in regard to clamps or quills, these, he says, are insufficient to hold a surface of from one to two inches in diameter, in apposition; and with the added danger of interrupted circulation from too firm pressure, he regards this method with less favor than the simple suture.

Admitting these defects the desideratum is a lateral force which shall approximate the denuded surfaces without antero-posterior force exerted on the tissues. In order to produce this he has invented an instrument which is described. The operation is made the same as ordinarily, and the sutures introduced as for the interrupted suture. The instrument which he now employs consists of nickel plated "stays." These are of about the diameter of a large surgeon's needle, and vary in length from $\frac{3}{4}$ to $2\frac{1}{4}$ inches, each extremity terminating in a fork, one of which is movable. After the introduction of the sutures one of these stays, a little longer than the antero-posterior depth of the surfaces, is introduced between the lips of the wound. and the extremity holding the movable fork fixed against that portion of the suture which shows itself in the vagina, the free ends of the sutures are then twisted over the external fork until the parts are adapted, and so on till all the sutures are thus secured. It will now be seen that the antero-posterior pressure is made upon the stay, and hence all corrugation prevented while the lateral power approximates the surfaces smoothly. In removing the sutures, after cutting them, the movable fork is held in the vagina with a pair of forceps and the stay pulled straight out, the movable fork is then brought out through the vulval orifice. No fistula has thus far resulted from the employment of these stays. The paper concludes with a report of four cases in which this instrument was employed with a favorable result, and a postscript gives a report of a like result in a fifth. In the fourth and fifth cases cat-gut sutures were employed, of which the author speaks highly.

